# Evaluation of the Combined Use of Saccharomyces Cerevisiae and Aspergillus Oryzae with Phytase Fermentation Products on Growth, Inflammatory, and Intestinal Morphology in Broilers

**DOI:** 10.3390/ani9121051

**Published:** 2019-12-01

**Authors:** Wen Yang. Chuang, Wei Chih. Lin, Yun Chen. Hsieh, Chung Ming. Huang, Shen Chang. Chang, Tzu-Tai Lee

**Affiliations:** 1 Department of Animal Science, National Chung Hsing University, Taichung 402, Taiwan; xssaazxssaaz@yahoo.com.tw (W.Y.C.); waynezi2227738@gmail.com (W.C.L.); richard840909@gmail.com (Y.C.H.); wxads508@gmail.com (C.M.H.); 2 Kaohsiung Animal Propagation Station, Livestock Research Institute, Council of Agriculture, Pingtung 912, Taiwan; macawh@mail.tlri.gov.tw; 3 The iEGG and Animal Biotechnology Center, National Chung Hsing University, Taichung 402, Taiwan

**Keywords:** *Saccharomyces cerevisiae*, *Aspergillus oryzae*, phytase, fermentation, broilers

## Abstract

**Simple Summary:**

The stress and anti-nutrient effect caused by environmental problems and animal feed is an urgent problem in poultry production. As ancient probiotics, *Aspergillus oryzae* and *Saccharomyces cerevisiae* can effectively improve the immunity of animals. Furthermore, the anti-nutrient object, phytate, reduces nutrition absorption. Therefore, *S. cerevisiae* or *A. oryzae* with phytase co-fermentation may help solve these problems. Results show that the addition of a fermentation product can effectively reduce the inflammatory response and drop the number of harmful bacteria in the ileum of broilers. Among them, *A. oryzae* fermentation product has a better effect than *S. cerevisiae* fermentation product.

**Abstract:**

*Saccharomyces cerevisiae* and *Aspergillus oryzae* are both ancient probiotic species traditionally used as microbes for brewing beer and soy sauce, respectively. This study investigated the effect of adding these two probiotics with phytase fermentation products to the broilers diet. Fermented products possess protease and cellulase, and the activities were 777.1 and 189.5 U/g dry matter (DM) on *S. cerevisiae* fermented products (SCFP) and 190 and 213.4 U/g DM on *A. oryzae* fermented products (AOFP), respectively. Liposaccharides stimulated PBMCs to produce nitric oxide to 120 μmol. Both SCFP and AOFP reduced lipopolysaccharides stimulated peripheral blood mononuclear cells (PBMCs) nitric oxide release to 40 and 60 μmol, respectively. Nevertheless, in an MTT (3-(4,5-dimethylthiazol-2-yl)-2,5-diphenyltetrazolium bromide) assay, SCFP and AOFP also increased the survival rate of lipopolysaccharides stimulated PBMCs by almost two-fold compared to the negative control. A total of 240 broilers were divided into four groups as Control, SCFP 0.1% (SCFP), SCFP 0.05% + AOFP 0.05% (SAFP), and AOFP 0.1% (AOFP) groups, respectively. Each group had 20 broilers, and three replicate pens. The results showed that the addition of SCFP, SAFP, and AOFP groups did not affect the growth performances, but increased the jejunum value of villus height and villus: crypt ratio on SAFP and AOFP groups compared to the control and SCFP groups. Furthermore, adding SCFP, SAFP, and AOFP significantly reduced the number of *Clostridium perfringens* in ileum chyme. SCFP, SAFP, and AOFP significantly reduced the amount of interleukin-1β, inducible nitric oxide synthases, interferon-γ, and nuclear factor kappa B mRNA expression in PBMCs, especially in the AOFP group. In summary, all the SCFP, SAFP, and AOFP groups can be suggested as a functional feed additive since they enhanced villus: crypt ratio and decreased inflammation-related mRNA expression, especially for AOFP group in broilers.

## 1. Introduction

As the global climate changes, the price of commodity feed ingredients, such as corn and soybean, rise continuously. Therefore, it is imperative to find new agricultural by-products as substitutes. Every year, millions of tons of wheat are produced all over the world, and wheat bran (WB) is the main by-product [1]. WB is inexpensive and may become one of the replacement sources for animal feed ingredients. WB consists of about 15% crude protein, 4% crude fat, 28% carbohydrate, 42% insoluble fiber, and is low in energy content. WB contains many compounds, such as ferulic acid, tocopherol, and lutein [2], which are good antioxidants. However, it contains high fiber content, 1% phytate, about 84% phytate-p and the total-p ratio [3] and susceptibility to mycotoxin contaminations because of improper storage. These have negative impacts on the utilization of WB as a viable ingredient in animal feed.

It is well-known that yeast cell walls are rich in ß-glucan and mannan, which can regulate immunity and protect the host from mycotoxins damage [4]. Although past studies have indicated that the addition of *S. cerevisiae* powder does not improve the growth traits of animals, it can increase the villi height and reduce the number of *E. coli* in the gut [5]. *S. cerevisiae* also has considerable enzyme secretion capabilities, such as proteases, xylanases, and cellulases [6]. In addition to *S. cerevisiae*, a previous study also pointed out that *A. oryzae*, generally recognized as safe (GRAS) probiotic, can be used as a probiotic for fermenting; it also has good enzyme secretion ability and can increase the crude protein content of the fermented material [7]. Moreover, many studies indicated that WB could increase its utilization value after fermentation by probiotics such as *Bacillus amyloliquefaciens*, *S. cerevisiae*, or white rot fungi in broilers diet [5,8]. Furthermore, a previous study pointed out that probiotics can decrease the inflammation response in animals [9]. Inflammation can cause many chronic diseases that affect animal health and longevity [10]. The reduction of the inflammatory response can decrease energy loss and improve the cell survival rate [11].

It is well-known that phytate possess high content of phosphorus and can chelate sodium, calcium or amino acids, thus affecting the absorption and utilization of nutrients by animals; however, phytase addition could solve the above-mentioned problem, and increase the digestibility of amino acids such as Lys, Met, Cys, and Thr [12], and the weight of the tibia, as well as increase the retention of phosphorus [13].

Few studies have been carried out on co-fermenting WB with probiotics combined with phytase to examine inflammation in broilers. Therefore, this study aims to investigate the *S. cerevisiae* fermented products (SCFP) and *A. oryzae* fermented products (AOFP) additions in broiler diets on the growth characteristics, intestinal morphology, microbial morphology, and inflammation-related mRNA expression in broilers.

## 2. Materials and Methods

### 2.1. Probiotic Characteristics

*S. cerevisiae* was cultured in Yeast-Mald (YM) broth at 30 °C for 0, 6, 12, 18, and 24 h, and then the number of *S. cerevisiae* colony-forming unit (CFU) on YM agar (Sparks, MD 21152 USA) was counted after sequence dilution to measure the growth curve of *S. cerevisiae. A. oryzae* was cultured in potato dextrose broth (PDB, Neogen, Lansing, MI, USA) at 30 °C for 0, 6, 12, 18, and 24 h, and the content of dry mycelia weight of *A. oryzae* in PDB was measured for the growth curve of *A. oryzae*. Moreover, 10^8^ CFU/mL *S. cerevisiae* or 10^6^ spores/mL *A. oryzae* fluid were incubated in 85 °C hot water for 3 min for heat tolerance measurement. After heating, the surviving *S. cerevisiae* and *A. oryzae* were counted on the YM agar or potato dextrose agar (PDA, Neogen, Lansing, MI, USA) respectively.

Acid, base, and gastrointestinal fluid tolerance were measured by pH-adjusted phosphate buffered saline (PBS, pH 3.0, 4.0, or 13.0, adjusted by citric acid or NaOH) (Merck, Darmstadt, Germany), simulation intestinal fluid (pH 13.0, 0.3% bile acid (Merck, Darmstadt, Germany), and 0.1% trypsin (Merck, Darmstadt, Germany)), or gastric fluid (pH 3.0, 0.3% pepsin). Here, 10^8^ CFU/mL *S. cerevisiae* or 10^6^ spores/mL *A. oryzae* fluid were incubated in the pH adjusted fluid, simulation intestinal fluid, or gastric fluid for 0 or 3 h at 30 °C. After incubating, the surviving *S. cerevisiae* and *A. oryzae* were counted on the YM agar or PDA, respectively.

The adherence ability was measured by the method indicated by Annika et al. [14], briefly, sacrificing the broiler and removing its crop. After dissection of the crop, sterile pH 7.2 PBS was used for rinsing. After washing, the crop epithelial cells were scraped with a slide and *S. cerevisiae* or *A. oryzae* spore solution was added. The state of attachment was confirmed under an optical microscope.

### 2.2. S. cerevisiae Fermented Products (SCFP) and A. oryzae Fermented Products (AOFP) Preparation and Characteristics

Then 10^8^ CFU/ mL of *S. cerevisiae* was added to dilute the fluid mentioned above into sterilized wheat bran at a ratio of 1 mL dilute fluid per 3 g of wheat bran. The moisture of *S. cerevisiae* pre-fermentation wheat bran was adjusted to 60%. After that, 500 phytase units (FTU) of phytase were added to 30 g *S. cerevisiae* pre-fermentation wheat bran. *S. cerevisiae* pre-fermentation wheat bran (contain phytase) was incubated at 30 °C for 5 days, and placed in a stove at 50 °C for 1 day. The *S. cerevisiae* fermented product (SCFP) was stored in a −20 °C refrigerator for the other test.

The spore counts of *A. oryzae* were diluted to 9 × 10^6^ spores/mL with deionized water. The diluted fluid was added into sterilized WB at a ratio of 1 mL dilute fluid per 3 g wheat bran. The moisture of *A. oryzae* pre-fermentation wheat bran was adjusted to 50%. After that, 500 FTU was added to 30 g *A. oryzae* pre-fermentation wheat bran. *A. oryzae* pre-fermentation wheat bran (contain phytase) was incubated at 30 °C for 7 days, and placed in a stove at 50 °C for 1 day. The *A. oryzae* fermented product (AOFP) was stored in a −20 °C refrigerator for the other test. The xylanase [15], protease [16], cellulase, and ß-glucanase [17] activities in SCFP or AOFP were analyzed before the animal experiment.

### 2.3. Peripheral Blood Mononuclear Cell Isolation

The assay was conducted and modified according to Kaiser et al. [18]. Briefly, 5 mL whole blood was collected from a total of thirty-six 35-d-old broilers (3 for each pen, 9 for each treatment) in the Summax Single-use Containers for Human Venous Blood Specimen Collection (Lithium Heparin Tube), and centrifuged at 200× *g* for 10 min to separate blood cells and plasma. The supernatant was replaced with sterilization PBS, and the sample was mixed uniformly. The mixed sample was added to the same amount of ficol, and centrifuged at 200× *g* for 30 min. The separated PBMC were moved to the new RNase free Eppendorf tube (Gunster Biotech, Co., Ltd., Taipei, Taiwan), and washed by PBS for three times. After this, the sample was centrifuged at 200× *g* for 10 min to remove the suspension PBS. RPMI-1640 (Merck, Darmstadt, Germany) with 10% fetal bovine serum (Merck, Darmstadt, Germany) (for cell test) or PBS (for qPCR) were used to re-suspend PBMCs and dilute it to 10^7^ cells/mL.

### 2.4. Nitric Oxide Assay

Isolation diluted PBMC (10^7^ cells/mL) were cultured at 37 °C and 5% CO_2_ for 2 h. Then 10 μL 1 ng/mL lipopolysaccharides (LPS) and 10 μL sample solution, which had been filtrated by the 0.22 μm filter, were added. Sterilized PBS was used as the control group. After co-incubation for 24 h, 100 μL Griess reagent was added, and the absorbance was detected at 540 nm.

### 2.5. 3-(4,5-Dimethylthiazol-2-yl)-2,5-Diphenyltetrazolium Bromide (MTT) Assay

Isolation diluted PBMC (10^7^ cells/mL) were cultured at 37 °C and 5% CO_2_ for 2 h. Then 10 μL 1 ng/mL lipopolysaccharides (LPS, Merck, Darmstadt, Germany) and 10 μL sample solution, which has been filtrated by 0.22 μm filter, were added. Sterilized PBS was used as the control group. After co-incubating for 48 h, 20 μL 0.5% MTT (Merck, Darmstadt, Germany) was added, and cultured for 4 h. After culturing, 100 μL dimethyl sulfoxide (DMSO, Merck, Darmstadt, Germany) was added, and the absorbance was detected at 570 nm.

### 2.6. Animal Experimental Design

To clarify the effect of SAFP, AOFP, and SAFP on broilers, the intestinal morphology, blood, and serum characteristics and mRNA expression in broilers’ PBMC were measured. This experiment was conducted at National Chung Hsing University, Taiwan. All of the protocols for animal use were followed by the Animal Care and Use Committee (IACUC: 107-013). The methods of animal design, intestinal morphology, blood and serum characteristics, and microbial parameter in intestinal content were slightly modified according to Teng et al. [5]. A total of 240 male one-day-old broiler chickens (Ross 308) were used and divided into four groups: Control, 0.1% SCFP (SCFP), 0.05% SCFP + 0.05% AOFP (SAFP), and 0.1% AOFP (AOFP), respectively. Each pen had 20 broilers, and 3 replicate pens (a total of 60 birds per group). The average body weight (42.0 ± 0.5 g/bird) was similar among every pen initially. All birds were placed in the temperature control house. At 0–3 day-old, the temperature was 33 ± 0.5 °C, and then the temperature decreased as the broiler grew up. Temperature was controlled at 22 ± 1 °C after 30 days. Diets were divided into starter and finisher (Table 1). Both of them met or exceeded the nutrient requirements of broilers (NRC, 1994) with addition of SCFP and AOFP. The proximate composition was analyzed according to the methods of Association of Official Analytical Communities (AOAC) [19]. The starter and finisher diets were offered to the birds from 1–21 day-old and 22–35 day-old respectively. Body weight and feed consumption were recorded at 21 and 35 day-old. Body weight gain and feed conversion ratios (FCR) were calculated. Nine birds of each group were randomly selected to isolate PBMCs for qPCR and cell test.

### 2.7. Intestinal Morphology

Twenty-four 35-day-old broilers (2 of each pen, 6 for each treatment, fasting for 1 day) were used for the measurement of intestinal morphology. The birds were euthanized, and the middle of the jejunum and ileum were excised, and the content was flushed by PBS and fixed in 10% formalin. Each sample was embedded by paraffin and stained with hematoxylin and eosin. The slice was observed with a light microscope. The villus height, crypt depth, and tunica muscular were measured by Motic Image Plus 2.0 analysis system (Motic Instruments, Richmond, BC, Canada).

### 2.8. Blood and Serum Characteristics

Thirty six 35-day-old broilers (3 of each pen, 9 for each treatment) blood was collected randomly for the blood and serum characteristic measurement. Blood samples stood for about 4 to 5 h at 4 °C, and then centrifuged at 3000 rpm for 10 min at 4 °C. Blood and serum biochemical parameters analyses were measured by automatic biochemical analyzer (Hitachi, 7150 auto-analyzer, Tokyo, Japan).

### 2.9. Microbial Parameter in Intestinal Content

Twenty-four 35-day-old broilers (2 of each pen, 6 for each treatment) were randomly selected for microbial analysis. Birds were euthanized, and the contents of ileum and cecum were squeezed out. Next, 1 g chyme was put into 9 mL PBS to sequence a dilution for the microbial parameter. *Lactobacillus* spp. were cultured with DeMan, Rogosa, and Sharpe agar (Difco™ Lactobacilli MRS Agar, BD, Franklin Lakes, NJ); *Clostridium perfringens* were cultured with tryptose sulfite cycloserine agar (GranuCult™ TSCagar, Merck KGaA, 64271, Darmstadt, Germany). Each microbe mentioned above was cultured at 37 °C under anaerobic conditions for 48 h. After culturing, the number of CFU on the agar was counted.

### 2.10. Total RNA Isolation and qPCR

Total RNA was isolated from PBMCs for determination of the mRNA expression level using SuperScript™ FirstStrand Synthesis System reagent (Thermo Fisher, Waltham, MA, USA) according to the manufacturer’s protocol. The method of determination of total RNA purity, cDNA synthesis, and qPCR analysis was slightly modified from Hu et al. [20]. Briefly, 2× SYBR GREEN PCR Master Mix-ROX (Gunster Biotech, Co., Ltd., Taipei, Taiwan), cDNA, deionized water, and each primer were mixed at a ratio of 5:1.2:1.8:1. StepOnePlus™ Real-Time PCR System (Thermo Fisher, Waltham, MA, USA) was used to detect qRT-PCR performance, while the 2^−△△Ct^ method was used to calculate the relative mRNA expression level, and ß-actin was used as the housekeeping gene for normalization. Gene-specific primers are according to the genes of Gallus gallus (chicken) given as follows: *beta-actin* (*ß-actin*, 5′-CTGGCACCTAGCACAATGAA-3′ and 5′-ACATCTGCTGGAAGGTGGAC-3′, X00182.1); *interlukin-1 beta* (*IL-1ß*, 5′-GCTCTACATGTCGTGTGTGATGAG-3′ and 5′-TGTCGATGTCCCGCATGA-3′, NM_204524); *inducible nitric oxide synthase (iNOS*, 5′-TACTGCGTGTCCTTTCAACG-3′ and 5′-CCCATTCTTCTTCCAACCTC-3′, U46504); *interferon-gamma* (*IFN-γ* (5′-CTCCCGATGAACGACTTGAG-3′ and 5′-CTGAGACTGGCTCCTTTTCC-3′, Y07922); *Nuclear factor kappa light chain enhancement of activated B cell p65 (NFκB p65*, 5′-CCAGGTTGCCATCGTGTTCC-3′ and 5′-GCGTGCGTTTGCGCTTCT-3′, D13719.1).

### 2.11. Statistical Analysis

Data were analyzed for significance by analysis of variance (ANOVA) using SAS software (SAS^®^ 9.4, 2018, SAS Institue Inc., Cary, NC, USA). Differences between treatment means were separated using Duncan’s multiple range test with *p* value less than 0.05.

## 3. Results

### 3.1. The General Characteristics of the Probiotics and Fermented Products

**Figure 1:**
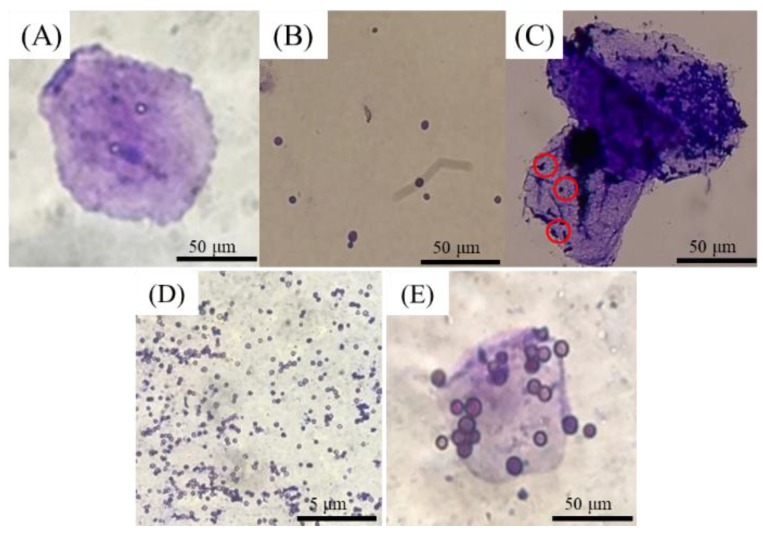
Photo of broiler crop epithelial cell (**A**), *Saccharomyces cerevisiae* (**B**), *Saccharomyces cerevisiae* adhering on the epithelial cell (red cycle) (**C**), spores of *Aspergillus oryzae* (**D**), and spores adhering on the epithelial cell (**E**), screened by an optical microscope.

After culturing for 24 h, the number of *S. cerevisiae* climbed to over 10^8^ CFU/g DM, while *A. oryzae* dry mycelia weight increased to 176.8 mg/100 mL PDB. Both *S. cerevisiae* and *A. oryzae* survived in acid, base solution, and even gastrointestinal fluid, but *A. oryzae* did not survive at 85 °C. Both *S. cerevisiae* and *A. oryzae* adhered to broiler crop epithelial cells. (Figure 1).

The results of the enzyme activity of *S. cerevisiae* fermented products (SCFP) and *A. oryzae* fermented products (AOFP) extraction are shown in Table 2. Wheat bran had no detectable enzyme activities; however, after fermentation by *S. cerevisiae* or *A. oryzae*, xylanase, protease, cellulase, and ß-glucanase all exhibited a dramatic increase. Fermentation by *S. cerevisiae* or *A. oryzae*, xylanase, protease, cellulase, and ß-glucanase increased significantly. The enzyme activities of SCFP were 142.26, 777.13, 189.49, and 117.07 U/g DM, respectively, and AOFP are 120, 190, 213.38, and 120.21 U/g DM, respectively. Compared to AOFP, SCFP had a higher protease (777.1 vs. 190.0 U/g DM) and xylanase (142.3 vs. 120.0 U/g DM) production ability. Furthermore, both SCFP and AOFP could produce cellulase and hemicellulase to degrade the fiber content in wheat bran.

**Figure 2:**
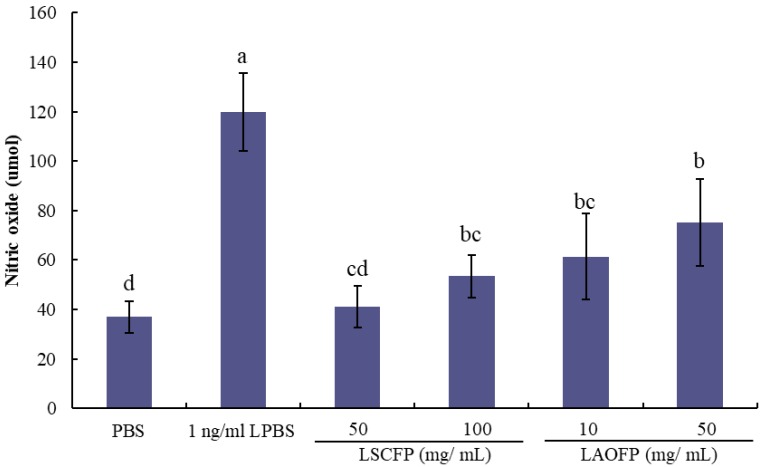
Nitric oxide release amount of phosphate buffer solution (PBS, control), 1 ng/mL lipopolysaccharide (LPBS, negative control), 50 mg/mL *Saccharomyces cerevisiae* fermented products extraction, and 1 ng/mL LPS include (50 mg/mL LSCFP), 100 mg/mL *Saccharomyces cerevisiae* fermented products extraction and 1 ng/mL LPS include (100 mg/mL LSCFP), 10 mg/mL *Aspergillus oryzae* fermented products extraction and 1 ng/mL LPS include (10 mg/mL LAOFP), and 25 mg/mL *Aspergillus oryzae* fermented products extraction and 1 ng/mL LPS include (25 mg/mL LAOFP). ^a,b,c,d^ Means within the same rows without the same superscript letter are significantly different (*p* < 0.05).

The results of nitric oxide (NO) release amounts are shown in Figure 2. After stimulating by LPS, the NO release amounts of PBMCs showed a significant increase. However, adding SCFP or AOFP extraction could reduce the NO release amount stimulated by LPS (*p* < 0.05). Among them, there were not significant differences in the 10 mg/mL LAOFP group, as well as the 50, 100 mg/mL LSCFP group, which means that AOFP has the same effect on reducing NO production of the LPS stimulated PBMC.

**Figure 3:**
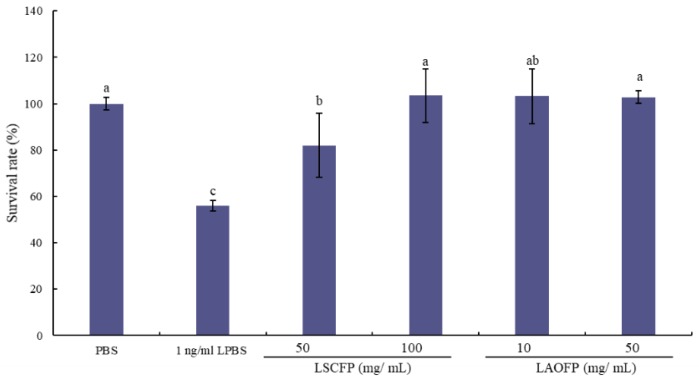
MTT (3-(4,5-dimethylthiazol-2-yl)-2,5-diphenyltetrazolium bromide) assay of phosphate buffer solution (PBS, control), PBS and 1 ng/mL lipopolysaccharide (LPBS, positive control), 50 mg/mL *Saccharomyces cerevisiae* fermented products extraction and 1 ng/mL LPS included (50 mg/mL LSCFP), 100 mg/mL *Saccharomyces cerevisiae* fermented products extraction and 1 ng/mL LPS included (100 mg/mL LSCFP), 10 mg/mL *Aspergillus oryzae* fermented products extraction and 1 ng/mL LPS include (10 mg/mL LAOFP), and 25 mg/mL *Aspergillus oryzae* fermented products extraction and 1 ng/mL LPS include (25 mg/mL LAOFP). ^a,b,c^ Means within the same rows without the same superscript letter are significantly different (*p* < 0.05).

As the results shown in Figure 3, the MTT assay has an opposite result of NO release amount on the data. LPS stimulates PBMCs and causes the survival rate of PBMCs to decrease significantly. However, adding SCFP or AOFP extraction could increase the PBMCs survival rate after stimulation by LPS (*p* < 0.05). Among them, there were not significant differences in the LAOFP group and 100 mg/mL LSCFP group, meaning that AOFP had the same effect on increasing the survival rate in the MTT test with a lower concentration.

### 3.2. Animals Performance

Growth performances of SCFP, SAFP, and AOFP supplemented are shown in Table 3. All treatments exhibited no significant differences on body weight, feed consumption, weight gain, and feed conversion ratio (FCR) at 1–21 days, 22–35 days, and 1–35 days.

**Figure 4:**
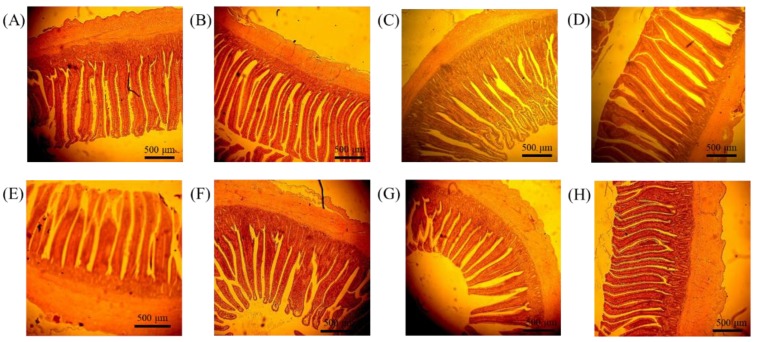
Photomicrography of jejunum and ileum of 35-day-old broiler feed with control, *Saccharomyces cerevisiae* fermented products (SCFP) or *Aspergillus oryzae* fermented products (AOFP). (**A**–**D**) jejunum, (**E**–**H**) ileum. Photos are respectively Control (**A**,**E**), 0.1% SCFP (**B**,**F**), 0.05% SCFP + 0.05% AOFP (**C**,**G**), and 1% AOFP (**D**,**H**). Hematoxylin and eosin stain 40×.

Although the results of growth performance showed no significant difference, the results of jejunum villus height and villus:crypt ratio on SAFP and AOFP both had a significant increase (1163.95 vs. 1508.93 and 1505.14 μm, 5.07 vs. 7.67 and 8.13, respectively, *p* < 0.0001). The crypt depth of the SCFP, SAFP, and AOFP groups showed a significant decrease (237.37 vs. 182.37, 203.43 and 188.42 μm, respectively, *p* = 0.0002). At the ileum, only SCFP and AOFP had a significant decrease on crypt depth (*p* < 0.0001), and AOFP had an increase in villus:crypt ratio (*p* = 0.0005) (Table 4). Photomicrography of jejunum and ileum of 35-day-old broilers are shown in Figure 4.

The white blood cells (WBC) increased significantly on SAFP (*p* < 0.0001) compared to other group. The concentration of uric acid (UA) significantly decreased following the treatment of SCFP and SAFP (*p* = 0.001). Furthermore, the SCFP group had a better effect on decreasing the UA content in serum compared to the AOFP group (3.36 vs. 4.69 mg/dL). However, there were no significant effects on triglyceride (TG), high-density lipoprotein (HDL), high-density lipoprotein (LDL), Ca, and P (Table 5).

**Figure 5:**
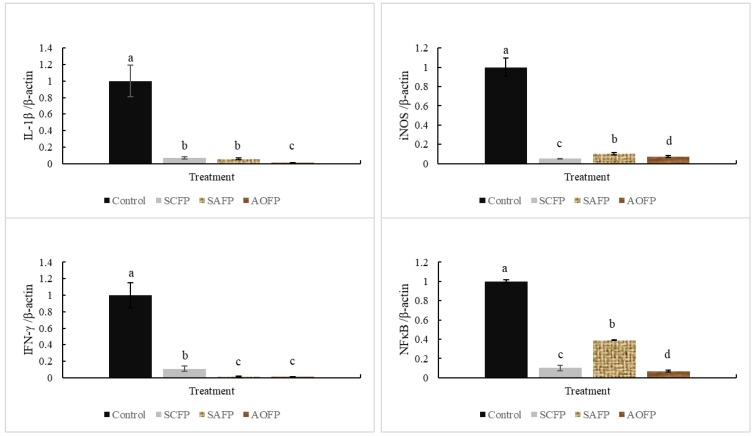
The mRNA expression level of immunomodulatory genes in chicken peripheral blood mononuclear cells at 35d. *IL-1ß* (top left), *iNOS* (top right), *IFN-γ* (bottom left), and *NFκB* (bottom right). The treatments are control, 0.5% *Saccharomyces cerevisiae* fermented products group (SCFP), 0.5% *Saccharomyces cerevisiae* fermented products + 0.5% *Aspergillus oryzae* fermented products group (SAFP), 0.5% *Aspergillus oryzae* fermented products group (AOFP), respectively. Data is presented in mean ± SE (n = 9). ^a,b,c,d^ Means within the same rows without the same superscript letter are significantly different (*p* < 0.05).

The results of mRNA expression level of immunomodulatory genes in chicken PBMCs are shown in Figure 5. As seen, the *IL-1ß*, *iNOS*, *IFN-γ*, and *NFκB* mRNA expression significantly decreased in SCFP, SAFP, and AOFP (*p* < 0.05). Furthermore, it is worth noting that AOFP can reduce the inflammation-related mRNA content most effectively.

### 3.3. Microbial Parameter in Intestinal Content

The treatment of SCFP, SAFP, and AOFP significantly decrease the number of *Clostridium perfringens* (*p* = 0.0014), but did not increase the number of *Lactobacillus* spp. (Table 6). In the caecum, although the number of *C. perfringens* did not significantly decrease (*p* = 0.6770), the number of *C. perfringens* still decreased in the data (7.39, 7.00, 7.01, and 7.22 log CFU/g in the Control, SCFP, SAFP, and AOFP groups, respectively), especially for the SCFP group.

## 4. Discussion

The high content of insoluble fiber and phytate could reduce the utilization of nutrition in animals. SCFP and AOFP contain quite a large amount of enzymes, such as cellulase, ß-glucanase, xylanase, and protease, thereby improving the nutrient utilization of broilers [21]. Nevertheless, adding phytase to the fermentation process of SCFP and AOFP may also help to reduce the phytate content [12]. From the results, the addition of SCFP and AOFP did not improve body weight, feed consumption, weight gain, and FCR. However, Santos et al. [22] reported that phytase addition (500 FTU/kg) improved the body weight gain of 1–21 day-old broilers, but did not improve the body weight gain and FCR in 22–35 day-old broilers. Mountzouris et al. [23] pointed out that *S. cerevisiae* powder (50 mg (10^9^ CFU)/kg diet) addition did not enhance the growth performance. Otherwise, there are few studies investigating the effects of *A. oryzae* addition on broilers growth performance. As a previous study shows, replacing soybean meal by *A. oryzae* fermented soybean meal (29.5% replacement in whole diet) can improve average daily gain and feed intake of broilers [24]. As the replacement is far higher than in the current study, it is speculated that the addition of *A. oryzae* should be sufficient to prove effective in growth performance.

The improvement of white blood cells (WBC) may be attributed to the joint effects of SCFP and AOFP because only SAFP increase WBC count in broilers blood. The SCFP and SAFP groups can significantly reduce the uric acid (UA) content in the blood, and the data number of SAFP is between SCFP and AOFP. This is because the protease yielded by SCFP was higher than AOFP and protease improved the protein utilization of the host, thereby decreasing the UA content in the blood. UA is a metabolite of protein that has an antioxidant function but is converted to a pro-oxidant in the cell or cytoplasm, and may be associated with cardiovascular disease when the concentration increases [25]. In addition, UA also promotes inflammation and is associated with insulin resistance and metabolic disorders [25]. Therefore, the addition of SCFP or AOFP to feed may prevent the development of cardiovascular disease, and reduce the oxidative stress of animal cells by the pathway of reduced UA.

In this study, villus height and the villus:crypt ratio in the jejunum were significantly increased and crypt depth significantly decreased on SAFP and AOFP, indicating that SAFP and AOFP may have an effect on improving nutrition absorption. One possible reason is that the enzyme yielded by SCFP and AOFP may degrade the anti-nutritional factor and make the energy utilization focus on villi growth and nutrient absorption [26]. Kalantar et al. [21] also reported that adding 0.1% commercial enzyme COMBO^®^ (including 1000 FTU/g phytase, 200 U/g xylanase, 200 U/g β-glucanase, and 80 U/g hemicellulase) in broilers diet can decrease the crypt depth and increase the villus length/width ratio and villus length/crypt depth ratio. Gao et al. [27] showed that yeast supplement (2.5, 5.0, and 7.5 g/kg diet) can increase the villus height of the small intestine. Feng et al. [24] found that replacing soybean meal by *A. oryzae* fermented soybean meal can increase villus height on both duodenum and jejunum and decrease the crypt depth in jejunum. In addition, a decrease in the crypt depth indicates that the animal can spend less energy on the formation of the crypt, thereby reducing the energy loss [26]. Furthermore, the results showed that Tunica muscularis decreased significantly on jejunum after adding SCFP, SAFP, and AOFP. Tunica muscularis consists of smooth muscle and is related to digestion and absorption of chyme. Cil et al. [28] reported that inflammation causes the intestine to swell and increases Tunica muscularis weight as well as thickness. Therefore, by the anti-inflammatory effect of SCFP, SAFP, and AOFP confirmed by cell test and broilers PBMCs mRNA expression, the decreased thickness of Tunica muscularis is expected.

Previous studies pointed out that probiotic can reduce the number of harmful microbes in the gut [5]. Mountzouris et al. [23] indicated that *S. cerevisiae* powder (50 mg (1 × 10^9^ CFU)/kg diet) can protect broilers from *Salmonella Enteritidis* (2 × 10^6^ CFU/birds) challenge at day 15 and increase the growth performance of broilers. In this study, adding SCFP, SAFP, and AOFP can reduce *C. perfringens* in the ileum. Among them, the SCFP is better than the AOFP based on the data, but with no significant difference, while the SAFP effect was in the middle. The antibacterial effect may be due to the growth of probiotics that crowd out the growth resources of *C. perfringens*, or the glucan and mannan produced by the probiotics encapsulate the toxins yield from *C. perfringens* and reduce the competitiveness of *C. perfringens*. Furthermore, the number of *C. perfringens* is positively correlated with the histochemistry score and necrotizing enterocolitis [29], thereby decreasing the number of *C. perfringens* may decrease the amount of pro-inflammatory cytokine.

LPS is an extracellular structure of gram-negative bacteria that can cause inflammation and stimulate the release amount of nitric oxide (NO) from PBMCs [11]. However, SCFP and AOFP can protect PBMCs from LPS stimulus and decrease the NO production. The reason may be that the glucan and mannan in *S. cerevisiae* and *A. oryzae* cell wall acted as antitoxin [3]. In addition, it is well-known that low-grade inflammatory response can protect the host from pathogens; however, high-grade inflammatory causes cell damage and apoptosis, so it is important to decrease the level of inflammation to prevent unexpected cell death. It is worth noting that AOFP has a similar effect as SCFP on lowering the concentration on NO production. Therefore, AOFP has similar effects as SCFP on lowering the concentration, which may represent that AOFP has a better anti-inflammatory effect than SCFP does.

For broilers, inflammation is a double-edged sword depending of its level [11]. Lee et al. [30] indicated that LPS challenge on broilers (1 mg/kg body weight) causes significant weight loss because of the immune response and decrease in feed intake. In broilers, there are two ways to decrease the inflammation level: one is to decrease the stimulants, such as LPS or bacterial toxin, and the other way is to block the pro-inflammatory cytokine pathway. From the results, SCFP and AOFP reduced the inflammation-related stimulant, including UA, *C. perfringens*, and NO production from LPS stimulated PBMCs.

As a pro-inflammatory cytokine, *IL-1ß* mediates many pathways involved in apoptosis or inflammation [31]. It is well-known that *S. cerevisiae* and *A. oryzae* cell wall are rich in β-glucans [3], which can inhibit *IL-1ß* production [32] and thereby reduce *NF-κB*-mediated inflammatory responses [33]. Therefore, *IL-1ß* is positively correlated with *NF-κB*. *NF-κB* is a main inflammatory factor, which promotes reactive nitrogen species (RNS) content [11]. In normal conditions, *NF-κB* binds with the inhibitory protein named inhibitor of kappa B (IκB) and has no effect [33]. With stimulants such as heat stress or infection, *IκB* phosphorylates and releases the *NF-κB* as a nuclear transcription factor to induce an inflammatory response [33]. SCFP, SAFP, and AOFP addition can decrease the *IL-1ß* and *NF-κB* mRNA expression. Hegazy and Bedewy [9] had similar results, indicating that 1 × 10^10^ CFU of *Lactobacillus delbruekii* and *L. fermentum* can decrease the interleukin-6 (IL-6), tumor necrosis factor-α (TNF-α), and *NF-κB* expression. The results show that probiotics reduced inflammation by suppressing the *NF-κB* and *IL-1ß* expression. Furthermore, the expression of *iNOS* can increase NO production, and is associated with infection [34], while SCFP and AOFP reduced the *iNOS* mRNA expression. *NF-κB* can induce *iNOS* expression, and increase the RNS production [35]; therefore, decreasing the *NF-κB* content can also suppress *iNOS* expression. *IFN-γ* is associated with infection and high concentration of *IFN-γ* may contribute to autoimmune disease [36]. When animals suffer from infection, *IFN-γ* expression increases and induces *NF-κB* expression to produce reactive oxygen species (ROS) and RNS to resist pathogens [37]. The data shown above indicate that SCFP and AOFP can decrease the damage caused by LPS and the number of *C. perfringens* in ileum, which are both related to the infection. Therefore, *IFN-γ* suppressed by SCFP, SAFP, and AOFP in broilers also decreases the *NF-κB* expression and reduces the inflammatory response [37]. Decreasing the stimulant and blocking the inflammation-related mRNA expression mentioned above confirm that both SCFP and AOFP addition can reduce the inflammation, especially AOFP.

## 5. Conclusions

Based on the above results, this study confirmed that SCFP, SAFP, and AOFP have positive effects on a decrease in *C. perfringens* number in ileum and suppress the mRNA relation of *IL-1ß*, *NF-κB*, *iNOS*, and *IFN-γ* on broilers’ PBMCs. Among them, SCFP, SAFP, and AOFP have different effects. Results showed that the addition of AOFP is much more effective than the addition of SCFP, while the effect of SAFP is somewhere between them, and there is no multiplication effect. Therefore, compared to the SCFP and SAFP, the AOFP is suggested to be a functional feed additive.

## Figures and Tables

**Table 1:** Composition and calculated analysis (g/kg as fed) of the basal diet for broilers (1–35 days) ^1^.

Ingredients	Starter Diet	Finisher Diet
	(1–21 Days)	(22–35 Days)
	g/kg	
Corn, yellow	485.5	535.6
Soybean meal, (CP 44 %)	348	281.4
Full-fat soybean meal	82.9	99.8
Soybean oil	36.5	42.4
Calcium carbonate	16.6	13.3
Monocalcium phosphate	18.2	16.3
_DL_-Methionine	1.97	1.33
_L_-Lysine-HCl	3.64	3.21
NaCl	3.88	3.81
Choline-Cl	0.83	0.79
Vitamin premix ^2^	0.99	1.03
Mineral premix ^3^	0.99	1.03
Total	1000	1000
Calculated nutrient value		
Dry matter, %	88.3	88.2
Crude protein, %DM	23	21
Crude fat, %	6.64	8.95
Calcium, %DM	1.05	0.9
Total Phosphorus, %DM	0.77	0.68
Available Phosphorus, %DM	0.5	0.45
Lysine, %DM	1.43	1.25
Methionine + Cysteine, %DM	1.07	0.96
ME, kcal/kg DM	3050	3175
Chemical analysis value		
Dry matter, %	88.0	88.7
Crude protein, %DM	23.1	20.9
Crude fat, %	6.58	8.78

^1^ Maize–soybean base diet for all treatment and add SCFP, SAFP, and AOFP to basal diet for SCFP, SAFP, and AOFP groups, respectively. ^2^ Vitamin (premix content per kg diet): vit. A, 15,000 IU; vit. D3, 3000 IU; vit. E, 30 mg; vit. K3, 4 mg; thiamine, 3 mg; riboflavin, 8 mg; pyridoxine, 5 mg; vitamin B12, 25 μg; Ca-pantothenate, 19 mg; niacin, 50 mg; folic acid, 1.5 mg; and biotin, 60 μg. ^3^ Mineral (premix content per kg diet): Co (CoCO_3_), 0.255 mg; Cu (CuSO_4_·5H_2_O), 10.8 mg; Fe (FeSO_4_·H_2_O), 90 mg; Mn (MnSO_4_·H_2_O), 90 mg; Zn (ZnO), 68.4 mg; Se (Na_2_SeO_3_), 0.18 mg.

**Table 2:** The xylanase, protease, cellulase, and ß-glucanase activities of SCFP and AOFP extraction (U/g DM).

Enzyme	Products		
	WB ^1^	SCFP ^2^	AOFP ^3^
Xylanase ^4^	ND ^5^	142.3	120.0
Protease ^6^	ND	777.1	190.0
Cellulase ^7^	ND	189.5	213.4
ß-glucanase ^8^	ND	117.1	120.2

^1^ WB: wheat bran. ^2^ SCFP: *Saccharomyces cerevisiae* fermented products. ^3^ AOFP: *Aspergillus oryzae* fermented products. ^4^ One unit of xylanase activity is defined as 1 μmol D-xylose generated from 10 mg/mL xylan in the condition of 37 °C and pH 5.5 in a minute. ^5^ Not detectable. ^6^ One unit of protease activity is defined as 1 μg L-tyrosine generated from 10 mg/mL casein in the condition of 40 °C and pH 7.5 in a minute. ^7^ One unit of cellulase activity is defined as 1 μmol, where reducing sugar is generated from 10 mg/mL CMC in the condition of 37 °C and pH 5.5 in a minute. ^8^ One unit of ß-glucanase activity is defined as 1 μmol, where reducing sugar is generated from 5 mg/mL ß-glucan in the conditions of 37 °C and pH 5.5 in one minute.

**Table 3:** Effect of SCFP, SAFP, and AOFP supplemented in diet on growth performance of 1–35 day-old broilers (n = 3).

Item	Treatments					
	Control ^l^	SCFP ^1^	SAFP ^2^	AOFP ^3^	SEM ^4^	*p* Value
1–21 days						
Body weight, g/bird	837.25	868.12	867.35	844.29	10.31	0.15
Feed consumption, g/bird	964.85	945.83	969.97	907.50	63.68	0.89
Weight gain, g/bird	794.25	825.12	824.35	801.29	10.32	0.15
FCR ^5^	1.15	1.09	1.12	1.07	0.08	0.88
22–35 days						
Body weight, g/bird	2163.24	2164.70	2228.59	2168.29	28.90	0.37
Feed consumption, g/bird	2267.14	1915.24	2078.81	1984.48	124.15	0.28
Weight gain, g/bird	1325.99	1296.58	1361.24	1324.00	28.10	0.49
FCR	1.71	1.48	1.53	1.50	0.11	0.48
1–35 days						
Feed consumption, g/bird	3231.99	2861.08	3048.78	2891.98	157.51	0.38
Weight gain, g/bird	2120.24	2121.70	2185.59	2125.29	28.90	0.37
FCR	1.49	1.32	1.37	1.33	0.08	0.45

^1^ SCFP: 0.5% *Saccharomyces cerevisiae* fermented products group. ^2^ SAFP: 0.5% *Saccharomyces cerevisiae* fermented products + 0.5% *Aspergillus oryzae* fermented products group. ^3^ AOFP: 0.5% *Aspergillus oryzae* fermented products group. ^4^ SEM: Standard error of the mean. ^5^ FCR: Feed conversion rate.

**Table 4:** Effect of SCFP, SAFP, and AOFP supplemented in diet on intestinal morphology of 35 d-old broilers (n = 6).

Item	Treatments					
	Control	SCFP ^1^	SAFP ^2^	AOFP ^3^	SEM ^4^	*p* Value
Jejunum						
Villus height (μm)	1163.95 ^b^	1124.78 ^b^	1508.93 ^a^	1505.14 ^a^	30.69	<0.0001
Crypt depth (μm)	237.37 ^a^	182.37 ^c^	203.43 ^bc^	188.42 ^c^	8.59	0.0002
*Tunica muscularis* (μm)	298.32 ^a^	285.47 ^b^	272.12 ^b^	255.92 ^b^	14.17	0.004
Villus:crypt	5.07 ^c^	6.36 ^b^	7.67 ^a^	8.13 ^a^	0.3	<0.0001
Ileum						
Villus height (μm)	1109.77	1055.33	1074	1050.13	19.04	0.1185
Crypt depth (μm)	209.25 ^a^	173.25 ^b^	201.29 ^a^	157.32 ^b^	6.82	<0.0001
*Tunica muscularis* (μm)	650.8	439.38	366.38	318.68	108.05	0.1453
Villus:crypt	5.52 ^b^	6.25 ^ab^	5.44 ^b^	7.08 ^a^	0.28	0.0005

^1^ SCFP: 0.5% *Saccharomyces cerevisiae* fermented products group. ^2^ SAFP: 0.5% *Saccharomyces cerevisiae* fermented products + 0.5% *Aspergillus oryzae* fermented products group. ^3^ AOFP: 0.5% *Aspergillus oryzae* fermented products group. ^4^ SEM: Standard error of the mean. ^a,b^ Means within the same rows without the same superscript letter are significantly different (*p* < 0.05).

**Table 5:** Effect of SCFP, SAFP and AOFP supplemented in diet on serum characteristics of broilers (35 d) (n = 9).

Treatments	Items ^1^							
	WBC	UA	CHOL	TG	HDL-C	LDL-C	Ca	P
Units	10^3^/uL	mg/dL	mg/dL	mg/dL	mg/dL	mg/dL	mg/dL	mg/dL
Control	189.69 ^b^	5.57 ^a^	119.22	77	73.44	38.33	9.86	9.13
SCFP ^2^	193.69 ^b^	3.36 ^c^	117.44	61.33	74.56	38.11	9.51	9
SAFP ^3^	234.46 ^a^	4.16 ^bc^	133.22	89.11	81.33	41.78	9.99	8.94
AOFP ^4^	185.40 ^b^	4.69 ^ab^	125.33	66.56	77.11	42.89	9.77	8.72
SEM ^5^	4.35	0.35	5.84	8.19	3.67	2.95	0.12	0.34
*p* Value	<0.0001	0.001	0.2359	0.1017	0.4479	0.576	0.0641	0.8596

^1^ WBC: white blood cell; UA: uric acid; CHOL: cholesterol; TG: triglycerides; HDL: cholesterol-high-density lipoprotein; LDL-C: cholesterol-low-density lipoprotein; Ca: calcium; P: phosphorus. ^2^ SCFP: 0.5% *Saccharomyces cerevisiae* fermented products group. ^3^ SAFP: 0.5% *Saccharomyces cerevisiae* fermented products + 0.5% *Aspergillus oryzae* fermented products group. ^4^ AOFP: 0.5% *Aspergillus oryzae* fermented products group. ^5^ SEM: standard error of the mean. ^a,b,c^ means within the same rows without the same superscript letter are significantly different (*p* < 0.05).

**Table 6:** Effect of SCFP, SAFP, and AOFP supplemented in diet on microbial parameter in the intestinal content of 35 d-old broilers (n = 3).

Microbial Parameter	Treatments					
	Control	SCFP ^1^	SAFP ^2^	AOFP ^3^	SEM ^4^	*p* Value
	log CFU/g					
Ileum						
*Clostridium perfringens*	7.84 ^a^	7.02 ^b^	7.05 ^b^	7.22 ^b^	0.12	0.0014
*Lactobacillus* spp.	8.35	8.84	8.25	8.91	0.39	0.1765
Caecum						
*Clostridium perfringens*	7.39	7.00	7.01	7.22	0.24	0.6770
*Lactobacillus* spp.	8.95	9.13	8.92	8.91	0.23	0.7039

^1^ SCFP: 0.5% *Saccharomyces cerevisiae* fermented products group. ^2^ SAFP: 0.5% *Saccharomyces cerevisiae* fermented products + 0.5% *Aspergillus oryzae* fermented products group. ^3^ AOFP: 0.5% *Aspergillus oryzae* fermented products group. ^4^ SEM: Standard error of the mean. ^a,b^ Means within the same rows without the same superscript letter are significantly different (*p* < 0.05).

## Abbreviations

SCFP	*Saccharomyces cerevisiae* fermented products
AOFP	*Aspergillus oryzae* fermented products
PBMC	Peripheral blood mononuclear cells
LPS	Lipopolysaccharides
SAFP	The mix of SCFP and AOFP at a ratio of 1:1;
WB	Wheat bran
GRAS	Generally recognized as safe
Lys	Lysine
Met	Methionine
Cys	Cysteine
Thr	Threonine
YM	Yeast-Mald
CFU	Colony-forming unit
PDB	Potato dextrose broth
PBS	Phosphate buffered saline
FTU	Phytase units
RPMI	Roswell park memorial institute
FBS	Fetal bovine serum
MTT	3-(4,5-Dimethylthiazol-2-yl)-2,5-Diphenyltetrazolium Bromide
DMSO	Dimethyl sulfoxide
MRS	Man, Rogosa, and Sharpe agar
TSC	Tryptose sulfite cycloserine
DM	Dry matter
NO	Nitric oxide
FCR	Feed conversion ratio
WBC	White blood cells
UA	Uric acid
TG	Triglyceride
HDL	High-density lipoprotein
LDL	High-density lipoprotein
*IL-1ß*	Interleukin-1 beta
*iNOS*	Inducible nitric oxide synthase
IFN-γ	Interferon-gamma
NFκB	Nuclear factor kappa light chain enhancement of activated B cell
RNS	Reactive nitrogen species
IκB	Inhibitor of kappa B
IL-6	Interleukin-6
TNF-α	Tumor necrosis factor-α
ROS	Reactive oxygen species
